# Supplementary material for: Nanofiltration as an Efficient Tertiary Wastewater Treatment: Elimination of Total Bacteria and Antibiotic Resistance Genes from the Discharged Effluent of a Full-Scale Wastewater Treatment Plant
Source: Antibiotics (Basel). 2022 May 6;11(5):630. doi: 10.3390/antibiotics11050630 (PMC9137456; doi:10.3390/antibiotics11050630)
Supplement: Supplementary file 1 [file antibiotics-11-00630-s001.zip › antibiotics-1700191-supplementary.pdf]

antibiotics

Table S1. Concentrations of total - live and dead - bacteria present in the discharged effluent, reused effluent, nanofiltered water and tap water samples. Values are expressed in cells per millilitre and correspond to the mean ± standard deviation of biological and technical triplicates.

	**Discharged effluent**	**Reused effluent**	**Nanofiltered water**	**Tap water**
Total bacteria	1.5E+06 (±5.5E+04)	8.0E+05 (±6.2E+04)	1.9E+04 (±2.7E+03)	8.8E+03 (±1.1E+03)
Live bacteria	1.1E+06 (±2.8E+04)	6.1E+05 (±4.4E+04)	1.3E+04 (±2.7E+03)	6.9E+03 (±8.2E+02)
Dead bacteria	4.0E+05 (±2.8E+04)	1.9E+05 (±1.8E+04)	6.2 E+03 (±1.2E+03)	1.8E+03 (±3.2E+02)

Table S2. Logarithmic reductions and removal rates of the total - live and dead - bacteria from the discharged effluent samples to the reused effluent and nanofiltered water samples.

	**Discharged effluent to reused effluent**		**Discharged effluent to nanofiltered water**	
	**Logarithmic reduction**	**Removal rate**	**Logarithmic reduction**	**Removal rate**
Total bacteria	0.28	47.02%	1.89	98.72%
Live bacteria	0.26	45.41%	1.93	98.82%
Dead bacteria	0.31	51.52%	1.81	98.44%

Table S3. Concentrations of the target carbapenem and (fluoro)quinolone resistance genes present in the DNA fraction of the discharged effluent, reused effluent, nanofiltered water and tap water samples. Values are expressed in gene copy numbers per millilitre and correspond to the mean ± standard deviation of biological and technical triplicates.

Gene	Discharged effluent	Reused effluent	Nanofiltered water	Tap water
bla_{KPC}	2.3E+04 (±2.9E+03)	5.5E+03 (±5.9E+02)	b.d.l.	b.d.l.
$bla_{OXA\text{-}48}$	1.4E+04 (±1.7E+03)	3.2E+01 (±1.1E+01)	b.d.l.	b.d.l.
bla_{NDM}	4.6E+03 (±1.2E+03)	b.d.l.	b.d.l.	b.d.l.
bla_{IMP}	1.1E+02 (±4.1E+01)	b.d.l.	b.d.l.	b.d.l.
bla_{VIM}	1.9E+05 (±2.4E+04)	8.8E+04 (±7.6E+03)	b.d.l.	b.d.l.
*qnr*A	8.1E+03 (±1.6E+03)	b.d.l.	b.d.l.	b.d.l.
*qnr*B	3.8E+04 (±1.3E+03)	2.2E+04 (±1.2E+03)	b.d.l.	b.d.l.
*qnr*S	5.9E+05 (±3.3E+04)	1.2E+05 (±6.7E+03)	b.d.l.	b.d.l.

b.d.l. below detection limit

Table S4. Removal rates of the target carbapenem and (fluoro)quinolone resistance genes from the discharged effluent samples to the reused effluent and nanofiltered water samples in the DNA fraction.

Genes	Discharged effluent to reused effluent	Discharged effluent to nanofiltered water
bla_{KPC}	75,83%	> 99.99%
bla_{OXA-48}	> 99.99%	> 99.99%
bla_{NDM}	> 99.99%	> 99.99%
bla_{IMP}	98,66%	> 99.99%
bla_{VIM}	53,97%	> 99.99%
*qnr*A	> 99.99%	> 99.99%
*qnr*B	42,81%	> 99.99%
*qnr*S	79,01%	> 99.99%

Table S5. Concentrations of the target carbapenem and (fluoro)quinolone resistance genes present in the eDNA fraction of the discharged effluent, reused effluent, nanofiltered water and tap water samples. Values are expressed in gene copy numbers per millilitre and correspond to the mean ± standard deviation of biological and technical triplicates.

Gene	Discharged effluent	Reused effluent	Nanofiltered water	Tap water
bla_{KPC}	b.d.l.	b.d.l.	b.d.l.	b.d.l.
$bla_{OXA\text{-}48}$	b.d.l.	b.d.l.	b.d.l.	b.d.l.
bla_{NDM}	b.d.l.	b.d.l.	b.d.l.	b.d.l.
bla_{IMP}	b.d.l.	b.d.l.	b.d.l.	b.d.l.
bla_{VIM}	1.3E+03 (±2.4E+02)	b.d.l.	b.d.l.	b.d.l.
*qnr*A	b.d.l.	b.d.l.	b.d.l.	b.d.l.
*qnr*B	b.d.l.	b.d.l.	b.d.l.	b.d.l.
*qnr*S	4.3E+02 (±2.3E+02)	2.8E+02 (±2.3E+02)	b.d.l.	b.d.l.

b.d.l. below detection limit

Table S6. Removal rates of the target carbapenem and (fluoro)quinolone resistance genes from the discharged effluent samples to the reused effluent and nanofiltered water samples in the eDNA fraction.

Genes	Discharged effluent to reused effluent	Discharged effluent to nanofiltered water
bla_{KPC}	-	-
bla_{OXA-48}	-	-
bla_{NDM}	-	-
bla_{IMP}	-	-
bla_{VIM}	> 99.99%	> 99.99%
*qnr*A	-	-
*qnr*B	-	-
*qnr*S	34.55%	> 99.99%

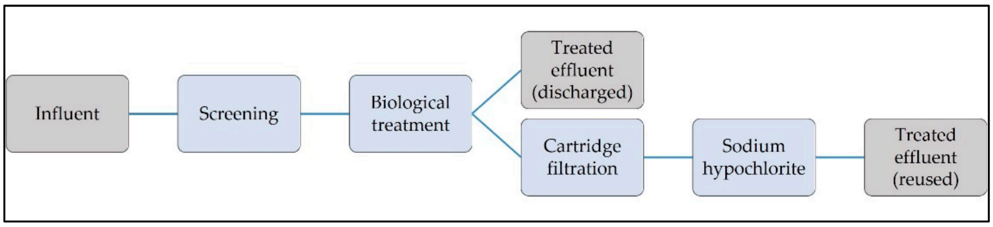

Figure S1. Main steps of the wastewater treatment applied in the full-scale WWTP selected for this study.

Table S7. General analytical control parameters of the discharged effluent samples collected for this study.

pH	TSS[1]	COD[2]	BOD_5[3]	Nitrogen	Nitrates	Phosporus	Chlorides	Coliforms
7.6	29 mg/L	110 mg/L O_2	25 mg/ O_2	32 mg/L N	4.1 mg/L N	1.972 mg/L P	180 mg/L Cl	1.30E+06 NMP/100 mL

[1] Total suspended solids

[2] Chemical oxygen demand

[3] Biological oxygen demand (5 days)
